# Normalized compression distance for DNA classification

**DOI:** 10.7717/peerj.20677

**Published:** 2026-02-06

**Authors:** Gavin Hearne, Mohammadsaleh S. Refahi, Haozhe (Neil) Duan, James R. Brown, Gail L. Rosen

**Affiliations:** Department of Electrical Engineering, Drexel University, Philadelphia, PA, United States of America

**Keywords:** Compression distance, Genomic sequence analysis, Genomic classification, Alignment-free methods, Bioinformatics, Metagenomics, Gzip compression

## Abstract

Analyzing the origin and diversity of numerous genomic sequences, such as those sampled from the human microbiome, is an important first step in genomic analysis. The use of normalized compression distance (NCD) has demonstrated capabilities in the field of text classification as a low-resource alternative to deep neural networks (DNNs) by leveraging compression algorithms to approximate Kolmogorov information distance. In an effort to apply this technique toward genomics tasks akin to tools such as Many-against-Many sequence searching (MMseqs) and Kraken2, we have explored the use of a *gzip*-based NCD combination in both gene labeling of open reading frames (ORFs) and taxonomic classification of short reads. Our implementation achieved 0.89 accuracy and 0.88 macro-F1 on human gene classification, surpassing similar NCD-based approaches. In prokaryotic gene labeling tasks, NCD shows superior classification accuracy to traditional alignment or exact-match tools in out-of-distribution settings, while also outperforming comparable sequence-embedding methods in in-distribution classification. However, the computational complexity of O(MN) (in standard big-O notation, where M and N denote the sizes of the training and test databases, respectively) constrains scalability to very large datasets, though these findings nonetheless demonstrate that compression-based approaches provide an effective alternative for genomic sequence classification, particularly in low-data environments.

## Introduction

The increasingly common use of next-generation sequencing has enabled far greater access to large-scale genomic and metagenomic datasets than ever before. Furthermore, advancements in processing techniques enable researchers to obtain unique biological insights into organisms without the need for improved wet-lab experiments, which are often time-consuming and expensive (Quince et al., 2017; Goodwin, McPherson & McCombie, 2016; Van Dijk et al., 2018). These advancements and the resulting deluge of data have made the quest for efficient DNA sequence search and classification methods, capable of identifying both taxonomic and functional elements from unknown sequences, an urgent challenge for all downstream analyses of sequencing methods.

Traditional sequence alignment-based methods struggle when presented with increasingly large volumes of sequence data due to the computational complexity of alignment (Zielezinski et al., 2019). Subsequently, there is a need for methods capable of sequence identification without alignment. One such example is k-mer frequency (Woloszynek et al., 2019; Rosen et al., 2011), which generates k-mer based representations of sequences which can then be searched for potential matches, avoiding excess computations at the expense of positional information. Alternatively, full nucleotide sequences can be represented as a book, with genes and other subsequence structures corresponding to chapters or paragraphs, words to k-mers, and letters to individual base pairs. This alternative characterization enables the use of natural language processing (NLP) to generate low-dimensional representations that can effectively capture more nuanced contextual information (Miller, Stern & Burstein, 2022; Detlefsen, Hauberg & Boomsma, 2022). State-of-the-art language models leveraging attention-based transformers (Dalla-Torre et al., 2025) and implicit convolutions (Nguyen et al., 2023; Nguyen et al., 2024) have demonstrated superior performance in diverse task sets, including classification and function prediction, due to their capacity to capture long-range genomic information. However, these models are complex and often require further training to achieve maximal performance in specific tasks (Brandes et al., 2022; Ji et al., 2021).

In this work, we explore the use of normalized compression distance (NCD) in genomic and metagenomic classification. NCD, when used in combination with K-Nearest-Neighbor (KNN) and the *gzip* file compressor, is a novel method of text classification developed by Jiang et al. (2023) based on the idea that texts of similar classes will have low information distance between them—that is, it would require few operations to convert one text to another and vice versa. Similar to NLP, in conceptualizing DNA sequences as text NCD-*gzip*-KNN can be directly applied towards genomic tasks without any adaptations to the underlying algorithm. A question remains: Can compression work well with the diversity of life, or is it best suited for genetic fragments that are highly similar? How does such an algorithm scale to the massive amounts of nucleotide data available? In this work, we adapt the NCD-*gzip*-KNN framework for genomic applications, extending it to handle multi-FASTA formats, large reference libraries, and tasks such as ORF labeling and metagenomic read classification. We then evaluate its effectiveness on several biologically relevant tasks, and clarify its computational tradeoffs in comparison to established tools.

## Related Works

### NCD with kernelization

Ali et al. (2024) extended the use of normalized compression distance for genomic classification by generating a kernel matrix from pairwise NCD values, which was then used with conventional classifiers such as SVMs, MLPs, and random forests. The kernelization step effectively embeds NCD into a machine-learning–friendly representation, but also introduces computational and memory overhead, since the full *N* × *N* kernel must be constructed and stored.

### Kraken2

Kraken2 is a common tool for alignment-free metagenomic read taxonomic classification, which built upon Kraken (Wood, Lu & Langmead, 2019) for enhanced speed and memory efficiency. This tool utilizes exact k-mer matching supplemented with a novel hash-table based database structure to enable extremely high-throughput classification speeds at high precision. As an exact k-mer match counter, there is a chance for zero matches to occur, leading to unclassified sequences. In these cases we consider those reads to not impact precision (they are not false positives); however, they do impact recall (they are false negatives). To get the highest number of hits, Kraken2 was set to match sequences with low confidence, meaning that sequences would be classified even with just a single k-mer match.

### MMseqs2

Many-against-Many sequence searching (MMseqs2) is another tool developed for sequence searching and clustering, utilizing a highly efficient seed-and-extend approach towards sequence alignment (Steinegger & Söding, 2017). Sequences are then classified using best-hit results.

### Sequence representation and retrieval

Representation learning, particularly through embedding sequences into multidimensional vectors, is essential for capturing complex patterns and relationships in genomic data. In this study, we employed two complementary techniques for generating embeddings: (1) 6-mer frequency vectors, which effectively capture local dependencies and sequence motifs, and (2) embeddings generated from genomic language models (GLMs), which incorporate higher-level positional encoding and are capable of modeling more complex, long-range sequence patterns. We selected recent GLMs (Nguyen et al., 2023; Refahi et al., 2025; Schiff et al., 2024) for their ability to handle long input sequences, enabling a fair comparison across embedding strategies. To identify the most similar matches for each embedding, we used Facebook AI Similarity Search (FAISS) (Johnson, Douze & Jégou, 2019), forming the basis for downstream retrieval and classification tasks.

### HyenaDNA

HyenaDNA (https://huggingface.co/LongSafari/hyenadna-medium-160k-seqlen-hf) (Nguyen et al., 2023) is a convolution-based model that replaces self-attention with implicit convolutions, enabling efficient processing of up to one million nucleotide tokens. Trained on the human reference genome at single-base resolution, it excels at modeling long-range dependencies for tasks such as regulatory element detection and species classification. Its sub-quadratic complexity allows faster training with high accuracy.

### MetaBERTa

MetaBERTa (https://huggingface.co/MsAlEhR/MetaBERTa-bigbird-gene) (Refahi et al., 2025; Refahi, Sokhansanj & Rosen, 2023) is a masked language model trained using 6-mer tokenization on a diverse set of bacterial and archaeal genes (Scorpio-Gene-Taxa dataset). It is built on BigBird (Zaheer et al., 2020), a sparse attention transformer architecture optimized for long sequences and originally applied to genomic inputs up to 4,096 tokens. MetaBERTa leverages this design to efficiently capture both functional and taxonomic signals, making it well-suited for gene function annotation, microbial classification, and gene clustering.

### CADUCEUS

CADUCEUS (https://huggingface.co/kuleshov-group/caduceus-ps_seqlen-131k_d_model-256_n_layer-16) (Schiff et al., 2024) is a state space model trained with masked language modeling on long genomic sequences (up to 131k bp) from the human genome. It incorporates reverse complement equivariance, allowing it to treat forward and reverse DNA strands identically. Despite its compact architecture, CADUCEUS outperforms larger transformer models on long-range genomic tasks such as variant effect prediction and regulatory region analysis.

## Methods

### Normalized compression distance

Compression distances have long been used as a metric for image and text similarity. These methods generally approximate Kolmogorov complexity (Kolmogorov, 1963) and information distance (Bennett et al., 1998), leveraging the power of compression algorithms to cluster data with similar information content (Cilibrasi & Vitányi, 2005).

Jiang et al. (2023) have developed such an approach for text classification by combining three components: a lossless compressor, a distance metric, and a K-nearest-neighbor (KNN) classifier. This method relies heavily on the concept of information distance as discussed by Bennett et al. (1998), defined as the shortest computer program to transform sequence *x* into *y*. This measure is expressed in terms of Kolmogorov complexity *K*(*x*), the length of the shortest program that produces *x* as its output:

(1) \begin{eqnarray*}E(x,y)=\max \nolimits \{ K(x{|}y),K(y{|}x)\} \end{eqnarray*}

(2) \begin{eqnarray*}=K(x,y)-\min \nolimits \{ K(x),K(y)\} .\end{eqnarray*}

Here, *K*(*x*|*y*) is the amount of information required to describe *x* given that *y* is already known, and *K*(*x*, *y*) is the complexity of the concatenation of *x* and *y*. Notably, true Kolmogorov complexity is by nature incomputable, and as such, information distance is instead approximated using compression distance *NCD*(*x*, *y*) as defined in Li et al. (2004):

(3) \begin{eqnarray*}NCD(x,y)= \frac{C(x,y)-\min \nolimits \{ C(x),C(y)\} }{\max \nolimits \{ C(x),C(y)\} } .\end{eqnarray*}

Here, *C*(*x*) and *C*(*y*) are the compressed lengths of encoded (in bytes) sequences *x* and *y*, found using the popular file compression software *gzip* (Deutsch, 1996). *C*(*x*, *y*) is the compressed length of their concatenated sequence. This technique is not limited to *gzip*, and any lossless compression algorithm may be utilized in this approximation.

The intuition is straightforward: similar sequences share repeated features. Repeated elements compress more readily than unique ones. After computing distances from a test sequence to the database, we apply KNN. The nearest hits determine the label. This method can be applied towards any text, and with a sufficiently large database has been shown to generate results that can rival other conventional classification methods and even large language models in text classification applications (Jiang et al., 2023).

**Figure 1:**
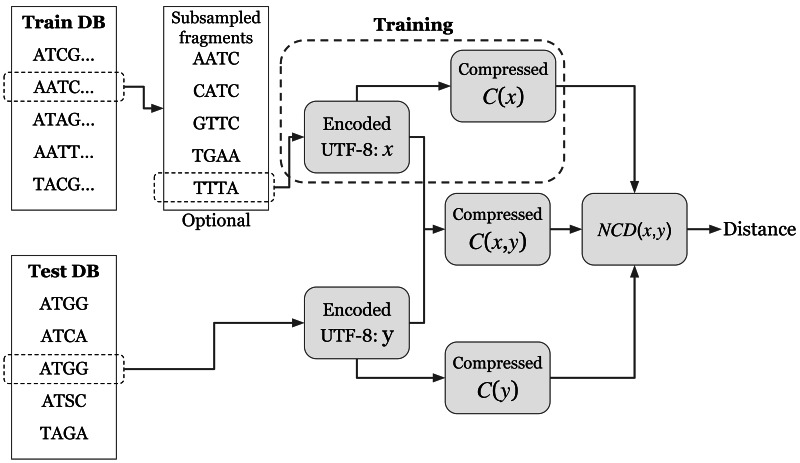
NCD process for a single comparison. First the Training and Testing sequences are assembled. Then for large sets, sequences are subselected to assemble a training database. The training and testing databases are encoded into UTF-8 to avoid nonstandard character representations then they are each compressed independently and jointly to compute the NCD. *The training process, the region marked with a dotted line, is precalculated for all training sequences, and does not need to be repeated for each testing sequence. ** C(y) is only calculated once for each testing sequence as well in the testing process.

Our usage is based off a framework developed by Schutte (2023a) and Schutte (2023b), the repository of whom can be found on GitHub (Schutte, 2023c). Schutte’s work notes and resolves several deficiencies in the original (Jiang et al., 2023) paper, primarily the incorrect calculation of KNN accuracy which resulted in inflated accuracy results, and redundant calculations in the NCD algorithm itself. Jiang’s original implementation calculated every compressed length of the training data for each testing sequence. These lengths can instead be precalculated (this we refer to as NCD training, shown in Fig. 1), resulting in a significant improvement in runtime (Schutte, 2023b). Our own architecture was then implemented to make this method compatible with multi-FASTA formats, for use in genomics research. To reduce memory consumption for larger tests such as metagenomic classification, which initially generated a large pairwise distance matrix, we included the real-time calculation of minimum distance hits to reduce this matrix size (and subsequent memory usage) significantly. However, there were no changes made to the base DEFLATE algorithm used by *gzip*, and all improvements do not impact the resulting compression ratios.

#### NCD timing

The primary limiting factor in computing NCD is the time it takes to compress files. Estimating this value is relatively simple, as it is a factor of several known quantities: *N*—the number of testing sequences, ${\bar {V}}_{testing}$—the average volume of data for each testing sequence, *V*_*training*_—the volume of data for the entire training database, and *R*—the compression speed of the compression algorithm when run on your hardware (*i.e.,* if it takes your system 20s to compress a 500 MB file, your compression speed is 25 MB/s. This is a speed that is relatively independent of data). The total time, *T*, can be calculated as:

(4) \begin{eqnarray*}T=N\times \left( {\bar {V}}_{testing}+{V}_{training} \right) \times 1/R.\end{eqnarray*}

For our system, the compression speed, *R*, is roughly 10 MB/s—this becomes an issue in particular with larger datasets, which can take multiple days to run.

### Datasets

This study uses three separate datasets of increasing complexity and size, sourced from human genes, prokaryotic (microbial) genes, and human microbiome metagenomes, a summary of which is shown in Tables 1 and 2.

#### Human DNA

Experiments reported in Ali et al. (2024) used NCD+*gzip* together with a kernel matrix generated from sequence representations. This combination achieved high accuracy on a small human DNA gene classification task, even outperforming state-of-the-art machine learning–based text classifiers such as BERT. The database used in these experiments consists of 4,380 sequences (each consisting of a single gene) and seven total gene classes from human DNA samples, and is broken into a 60-10-30 training, validation, testing split for 5-fold validation.

#### RefSeq prokaryotic genes

This is a 587 mb database built from prokaryotic genomic (DNA) data extracted from the RefSeq database utilizing Woltka’s pipeline (Zhu et al., 2022), downloaded in July 24, 2023. Initially containing data representing a single whole genome from every genera, this was reduced to only Bacteria/Archaea genomes. Full genes were then extracted and filtered to remove unknown and hypothetical proteins, as well as outliers in representation (by removing genes with fewer than 1,000 instances). From this, a testing set was built with three different conditions: Taxa_out (12 MB, 11K reads), gene_out (48 MB, 50K reads), test (150MB, 140k reads). Taxa_out contains no shared taxonomy (at phylum and lower) between the test and training datasets, Gene_out contains no repeated genes, and test contains both repeated genes and taxa (down to the family level). Ultimately, this contains 800,318 total sequences with 497 genes represented from 1,929 genera.

**Table 1:** Database sizes: # of sequences, file size, average length of sequence for training and testing. The added spacing clarifies which test set corresponds to which training set.

Dataset	Train			Test		
	Seq #	Size (MB)	Avg. Len	Seq #	Size (MB)	Avg. Len
**Human DNA** (Ali et al., 2024)	2,629	3.33	1,264.6	1,314	1.67	1,264.6
**Prokaryotic RefSeq Genes** (Refahi et al., 2025)	547,523	588	1,062.3	11,800^a^ 43,417^b^ 140,524^c^	12.51 48.16 150.66	1,061.3
**Metagenomic** (Duan et al., 2024)	3,708	11,610	2,225,712	92,599	13.54	126

**Notes.**

a Taxa_out test set.

b Gene_out test set.

c Full_test set.

**Table 2:** Database class counts across taxonomic levels for each dataset.

Level	Number of Classes							
	Human Genes		Prokaryotic RefSeq Genes				Metagenomic	
	Train	Test	Train	Test	Taxa_out	Gene_out	Train	Test
Superkingdom	1	1	2	2	1	2	4	4
Phylum	1	1	27	27	18	27	83	55
Class	1	1	83	83	25	83	176	103
Order	1	1	204	204	27	204	336	188
Family	1	1	505	505	33	505	829	369
Gene	7	7	437	437	436	60	–	–

#### Metagenomic reads

This dataset utilizes same initial setup as the RefSeq Prokaryotic Genes set. Woltka’s RefSeq build.py script (Zhu et al., 2022) was used to extract genomic (DNA) data from RefSeq, with data selected such that every genus contains a single genome reference, resulting in a total of 4,634 full genomes, including those from Archaea, Bacteria, Eukaryota, and Viruses superkingdoms. This was then randomly broken into a training/testing split of 926/3708 genomes. To simulate a metagenomic sample for testing purposes, the HiSeq InSilicoSeq model (Gourlé et al., 2019) was used to synthesize 100 reads of length 126 from each class, based on the selected testing genomes. For more details on this dataset, see Duan et al. (2024).

### Metagenomic training data subsampling

With NCD, it is important to consider the impact of sequence length on the distance calculation. Although not immediately obvious analytically, in practice two sequences with vastly different lengths tend to appear more distant than two with similar lengths (all else equal). In classifying metagenomic reads, there is both a large difference in length between the read and the full genomes, and between the genomes themselves. This means that it is important to normalize the length between the training database sequences, which can be achieved through fragmentation as described in the following ‘Genome fragmentation’ and ‘Taxa level fragmentation’. To perform genome-level classification from these fragments we compare two methods: first is by simply classifying to the genome of the minimum distance fragment (minimum hit), and second is by averaging the read-fragment distances within each genome, and subsequently classifying based on the genome with the minimum average distance (average genome).

#### Genome fragmentation

The most basic method of normalizing the lengths of the database genomes to both the testing reads and each other is achieved by splitting each genome into fragments of length 126 (the same as the testing reads). Then each fragment is compressed with the testing read, as opposed to each full sequence. This has two effects: first this allows for more useful values in the compressions. Compressing very small reads with significantly longer genomic reads will result in distances very near to 1, which are difficult to compare. Second, it allows for distances to be compared amongst genomes, as the length of each genome will no longer have an impact on the compression distance.

**Figure 2:**
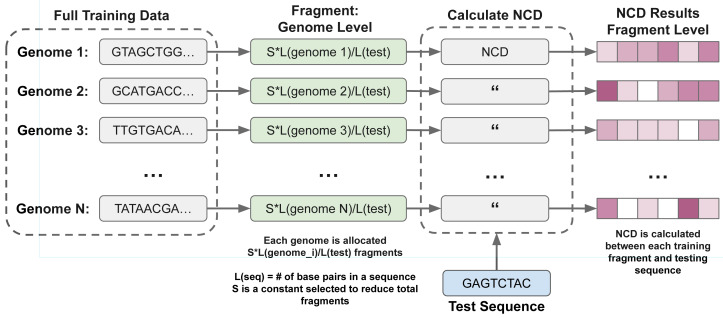
Allocation of training fragments per genome for genome-fragmentation. Each training genome is split into read-length fragments (length 126), with *K* randomly selected per genome proportional to *L*(genome_*i*_)/*L*(test). This fragmentation was used in metagenomic experiments in ‘Results’.

As a method of reducing the number of calculations necessary, a second implementation of gene fragmentation is also implemented. Rather than fragmenting each genome fully, we randomly select fragments from each genome, as shown in Fig. 2. Each genome is allocated *K* randomly selected fragments, where: $K=[ \frac{L(genom{e}_{i})}{L(test)} ]\ast S$. *S* is a constant, selected beforehand to reduce the number of fragments, *genome*_*i*_ is the *i*th genome in the database. This offers the promise of reducing total run-times at the cost of thoroughness.

#### Taxa level fragmentation

**Figure 3:**
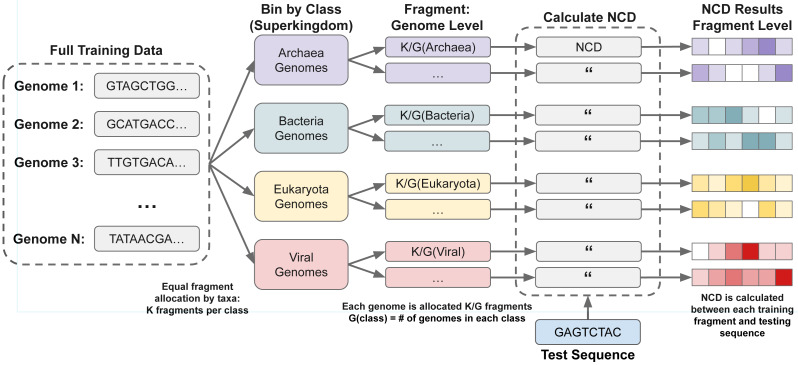
Taxa-rank balanced subsampling for metagenomic training. To mitigate class-volume imbalance, we sample an equal number of fragments per superkingdom. This strategy is used in the “Superkingdom Subsampling” experiments in ‘Results’.

However, fragmenting per-genome induces class-volume imbalance (longer genomes contribute more fragments and thus more opportunities for near-distance hits). To account for this effect, it is possible to instead select equal amounts of fragments from a taxa level to balance the classes. First a desired taxa level is selected, then the genomes in each class of that level are combined together. *K* fragments are then randomly selected from each combined class equally, resulting in classes with equal numbers of fragments, so no class with more or larger samples can overpower the smaller or shorter ones. The full process is shown in Fig. 3.

### Evaluation protocol

For consistency, each of the tools in ‘Related works’ (including *gzip*-NCD) are trained and tested on the same data (section ‘Datasets’) in each of the following task subsections. Also in each section, different classifiers are compared by macro F1, a standard multiclass classification metric which we elected to use due to the imbalanced nature of our data, which contained classes of vastly different quantities. Notably, there are two ways to compute macro-F1 that yield non-equivalent results (Opitz & Burst, 2019). Throughout, “macro-F1” denotes the “Averaged F1: arithmetic mean over harmonic means” definition in Opitz & Burst (2019).

### Hardware

All computations were performed on a computer cluster utilizing Dell PowerEdge R640 systems, each equipped with 2x Intel^®^ Xeon^®^ Platinum 8268 2.9 GHz 24-core processors and 192GB RAM.

## Results

### Human DNA

Previous research has demonstrated that the use of NCD+*gzip* along with a kernel matrix (Ali et al., 2024) is capable of achieving good performance in human gene classification. After averaging the results across five runs, this resulted in an accuracy up to 0.831 and a macro F1 of up to 0.813 as shown in Table 3. Our own analysis of the dataset, using the basic NCD+*gzip* with a minimum-distance classifier—in which each test gene is classified to its minimum-distance hit in the training database—, proved similarly competent across five runs on this database, achieving accuracy of 0.89 and macro F1 of 0.883.

Although this dataset is particularly simple, with few classes and highly similar conserved sections, this result demonstrates the viability of this method in gene classification. Empirically, our simpler NCD+*gzip* +KNN pipeline matched or exceeded kernel-based results while avoiding the substantial memory overhead of constructing and storing an *N* × *N* kernel. This outcome motivated our choice to proceed without kernelization in the remainder of our genomic applications.

### Prokaryotic gene classification

Classifying the gene class and taxonomy of prokaryotic genes can offer a much more clear picture into the performance of NCD in genomic applications. The inclusion of both gene and taxonomic classifications, as well as more testing scenarios clearly shows where a method such as NCD can perform best. Comparisons with six other classifiers are presented: 6-mer frequency, MetaBERTa (Refahi, Sokhansanj & Rosen, 2023; Refahi et al., 2025), HyenaDNA (Nguyen et al., 2023), CADUCEUS, MMseqs (Steinegger & Söding, 2017), and Kraken2 (Wood, Lu & Langmead, 2019).

#### Gene/taxa out

**Figure 4:**
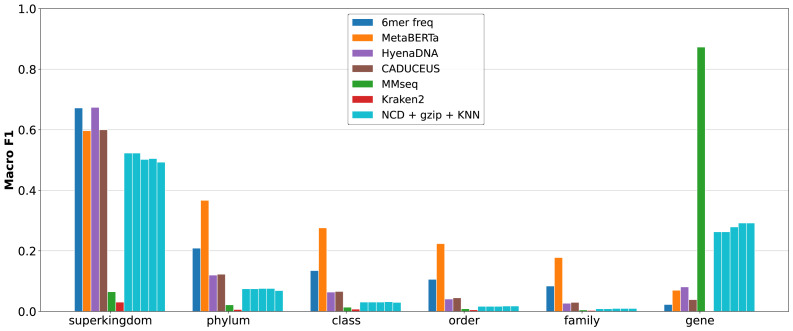
Macro-averaged F1 on held-out settings using the RefSeq Prokaryotic Genes dataset (‘Datasets’). Taxonomy is evaluated in the gene_out condition (no shared genes), and gene classification in the taxa_out condition (no shared taxa). Methods include NCD-*gzip* +KNN (*K* = 1 to 5, from left to right), 6-mer frequency, GLM-based embeddings (MetaBERTa, HyenaDNA, CADUCEUS) with FAISS retrieval, MMseqs2, and Kraken2 (taxonomy only). NCD+*gzip* offers the most balanced performance overall.

Withholding representative classes presents a difficult problem for metagenomic classifiers. When presented with either novel taxa or genes, traditional classifiers struggle, often being unable to classify either taxa or genes accurately. In the following Fig. 4, taxa classifications are performed using the gene_out dataset, and gene classifications are performed on the taxa_out dataset.

**Table 3:** Five-fold averaged results on the Human DNA gene dataset (Table 1). Reported metrics are mean across five runs. NCD+*gzip* (ours) uses minimum-hit classification. The NCD kernel-matrix baselines are from Ali et al. (2024). Results show that our base implementation outperforms the similar NCD+*gzip* +kernel matrix implementation across all metrics.

Method	Algo	Acc.	Prec.	Recall	F1 (Macro)
NCD+*gzip* + Kernel Matrix (Ali et al., 2024)	SVM	0.692	0.844	0.692	0.692
	NB	0.464	0.582	0.464	0.472
	MLP	0.831	0.833	0.831	0.813
	KNN	0.773	0.792	0.773	0.768
	RF	0.810	0.858	0.810	0.811
	LR	0.621	0.822	0.621	0.581
	DT	0.648	0.651	0.648	0.624
NCD+*gzip* (ours)	Min hit	**0.889 ± 0.0113**	**0.886 ± 0.0113**	**0.889 ± 0.0113**	**0.883 ± 0.0117**

**Notes.**

Results for NCD+gzip are shown in bold.

Traditional methods succeed for some specific tasks—*i.e.,* 6-mer frequency for taxa classification (although performance is lower than MetaBERTa), and MMseq for gene classification. However, this appears to be some degree specialized. While 6-mer frequency achieves high macro F1 in Gene_out classification, it is among the worst in Taxa_out, and vice versa for MMseqs. It is important to note that while Kraken2 does poorly on these tasks, this is not unreasonable. Kraken2 is a pure taxonomy classification tool optimized for speed, and performs best on larger datasets. GLM based representation learning methods show some superior generalization performance. In particular, MetaBERTa leverages the Bigbird model effectively to achieve best-in-set performance for taxonomy classification. However, this strong generalization performance is hampered by much lower performance in gene classification. It clear that while some GLMs are capable of effectively extracting high-level taxonomic features, they may be outperformed by raw sequence alignment in situations where taxonomic context is missing but gene similarity remains detectable. Regardless, the overall generalization of these learned models can be strong, which can be seen in the smaller performance gap between Taxa_out and Tene_out results relative to the traditional classifiers.

Impressively, NCD+*gzip* +KNN demonstrates a far superior ability to generalize to both Taxa_out and Gene_out than all other classifiers. Despite not the top performer in either task, NCD is capable of both detecting taxonomic signals without the need for representative genes, and properly identifying gene class from disparate taxa. This can be attributed to the complex nature of compression algorithms, which are capable of identifying and compressing both small and large features shared between two sequences. When compared to MMseqs2 and Kraken2, NCD+*gzip* +KNN is able to outperform their macro F1 scores by 2 to 8 times in taxa while outperforming representation learning methods by several times on gene classification. These results highlight the capability of NCD to perform as highly effective middle ground solution to both problems simultaneously.

#### Test

**Figure 5:**
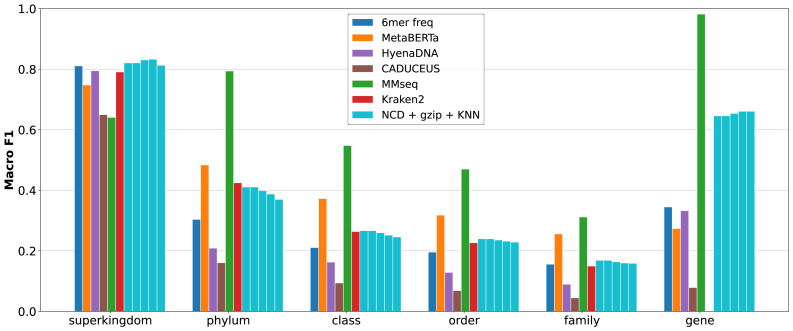
Macro-averaged F1 on the RefSeq Prokaryotic Genes test split containing representative genes and taxa (‘Datasets’). Bars include NCD-*gzip* +KNN (*K* = 1 to 5, from left to right), 6-mer frequency, GLM-based embeddings (MetaBERTa, HyenaDNA, CADUCEUS) with FAISS retrieval, MMseqs2, and Kraken2 (taxonomy only). NCD+*gzip* again offers the most balanced performance overall, and the best performance on superkingdom-level taxa.

The “test” dataset, containing both representative genes and taxa, proves to be ideal conditions for MMseqs2 to succeed, resulting in its performance far outmatching the other methods, shown in Fig. 5. It is clear that with suitable representation, MMseqs2 is the best across all taxonomic ranks while continuing to dominate gene classification. However, NCD does demonstrate clear advantages over all tools, MMseqs2 excluded.

In taxonomic classification, NCD+*gzip* performs on par with Kraken2 at all taxa ranks, while outperforming 6-mer at higher ranks. For gene classification, it is notable that excluding MMseqs2, no other tools come close the performance of NCD at any KNN values. An interesting observation in both the withheld and well-represented testing suites is the decrease in F1 as more nearest neighbors are accounted for in taxa classification, but higher performance in gene classification. This is likely a result of imbalanced database representation: the gene classes are equally balanced, whereas the taxonomic ones are not. The result being that at higher K values, those taxa with higher database representation have a greater chance to classify.

### Metagenomic read classification

The most challenging task that NCD is faced with is metagenomic taxonomy classification. The challenges are threefold. First, the volume of data is large: each read must be compressed against far more data than in the gene-classification setting. Second, the database is diverse in both size (requiring fragmentation) and taxonomy (the test set contains no representative genera, while including difficult viral and eukaryotic reads). Third, many reads contain regions not represented in the database, which makes them hard to classify

#### Genome fragmentation/subsampling

**Figure 6:**
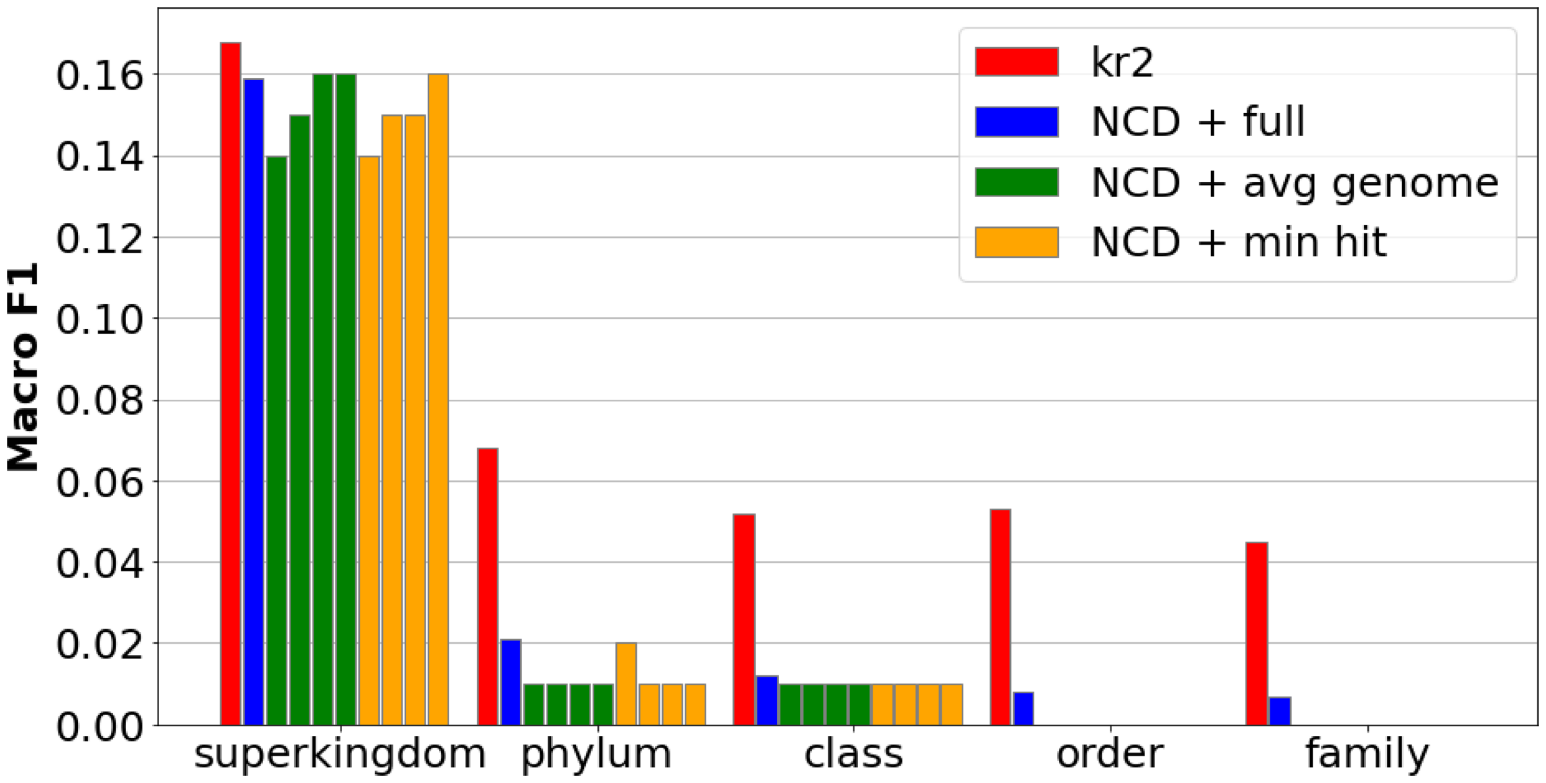
Macro-averaged F1 for metagenomic read taxonomy using genome fragmentation with random subsampling. Training: 3,708 RefSeq genomes fragmented to 126 bp (‘Genome fragmentation’); Repeated bars (from left to right) represent the following reduction in fragments-per-genome: 1/1,500, 1/3,000, 1/4,000, 1/5,000. Testing: 92,600 simulated reads (length 126) from the metagenomic dataset (‘Genome fragmentatio’). Kraken2 produces the highest classification accuracy overall. Subsampling reduces runtime but lowers performance relative to full genome fragmentation.

Breaking each training genome into a large number of equivalent length fragments removes the impact that training genome length has on the data (if this is not performed, almost all distances are near 1, with the exception of some particularly short virus genomes with only a few hundred base pairs). The second implementation of genome fragmentation, which includes subsampling to reduce the amount of data needed to be compressed, demonstrated poor capabilities in general. The results of both these methods are shown in Fig. 6.

**Figure 7:**
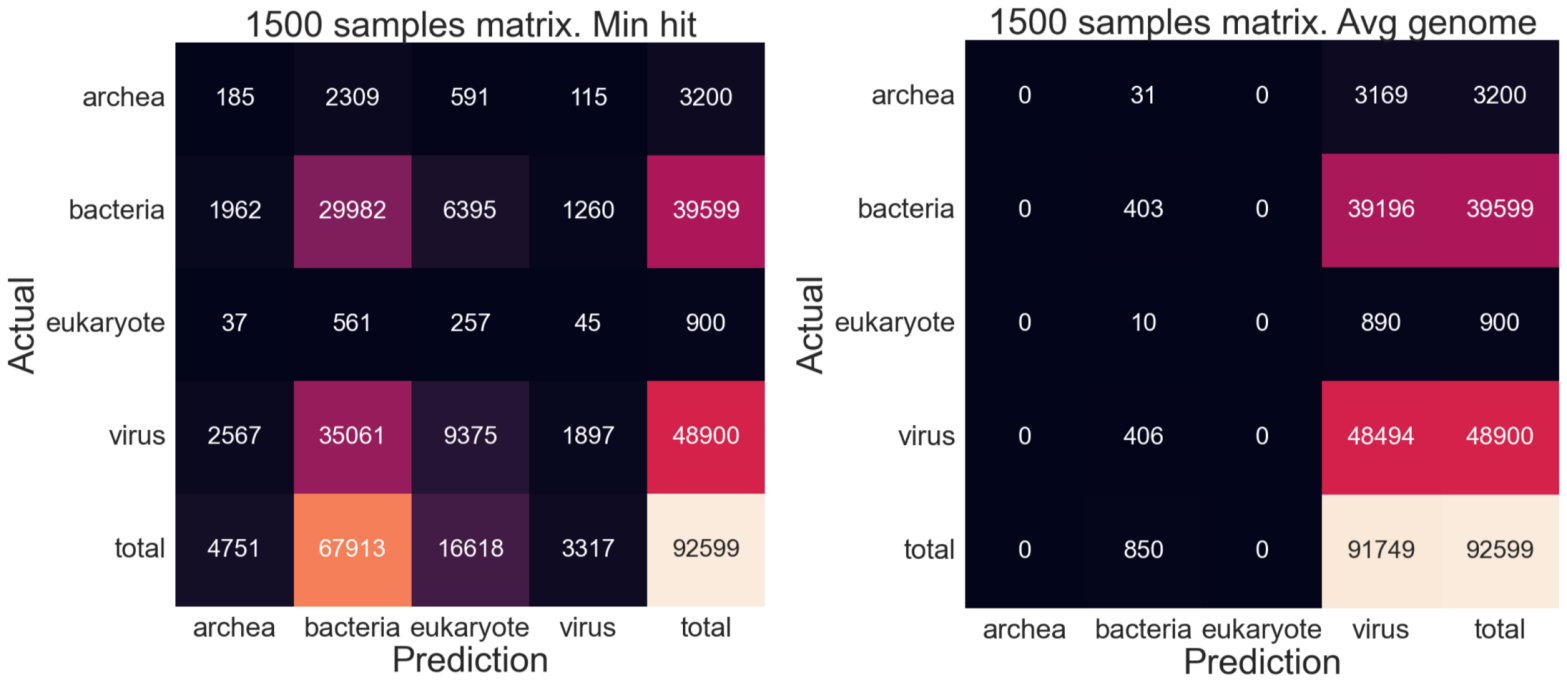
Confusion matrices for metagenomic taxonomy with genome-fragment subsampling at 1/1,500. Left: minimum-hit classification (assign read to the class of its nearest fragment). Right: averaged-genome classification (average read–fragment distances within each genome, then assign to nearest genome). Training and test data as in Table 1. The “total” column shows true class counts. Imbalanced fragment volumes skew predictions under different classification schemes.

In macro F1, genome subsampling falls behind Kraken2 in all metrics at ranks lower than superkingdom. The reason for this is clear: the imbalances in the volume of data for each upper rank class results in some classes having more representatives than others and thus overall, most classes have poor representation with the well-represented classes performing much better than the ill-represented ones. The effects of imbalanced database classes can be illustrated clearly in the resulting Fig. 7 confusion matrices.

For the minimum hit classification, prokaryote and eukaryote guesses dominate due to their longer lengths, enabling a far higher chance of randomly achieving a low-distance read, while virus guesses (which comprises of nearly half of the test reads) disappear almost entirely. The opposite effect can be observed when classifying by average genome, as virus guesses dominate due to their short lengths, due to outlier reads asserting more influence on sequences with few reads, while those with longer reads regress towards a mean.

#### Superkingdom subsampling

**Figure 8:**
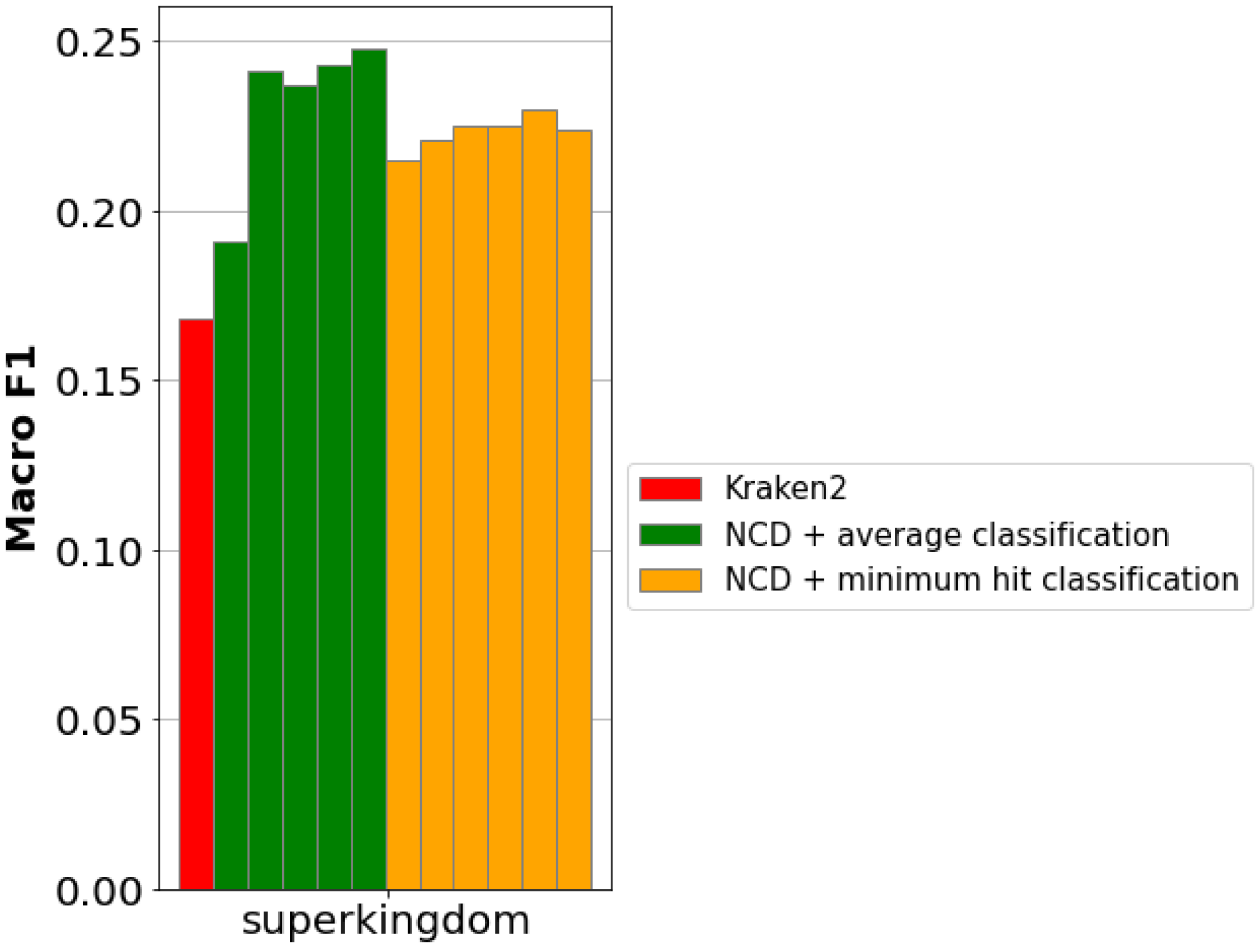
Macro-averaged F1 for metagenomic taxonomy with superkingdom-balanced subsampling. Training uses an equal number of fragments per superkingdom (100–2,500 per class). Testing uses the metagenomic read set (92,600 reads; length 126; ‘Metagenomic reads’). Repeated bars shown are (from left to right) the following number of samples per class: 100, 500, 1,000, 1,500, 2,000, 2,500. Balancing fragments per superkingdom yields competitive macro-F1, even with few samples.

By allocating each superkingdom an equivalent number of fragments, we can negate the effects of imbalanced class data volumes. While this does make it impossible to classify reads to a lower taxonomic rank than superkingdom, it can provide a look into the high level composition of a sample at far greater speeds than other NCD implementations. Multiple runs were performed to more clearly characterize the performance of this method, and the potential impacts the random fragment selection can have on the classification accuracy, shown in Fig. 8.

As with genome subsampling, two methods for hit classification were considered: minimum hit (each read is classified to the same class as the fragment with the nearest distance) and average (reads are classified to the superkingdom with nearest average distance).

**Figure 9:**
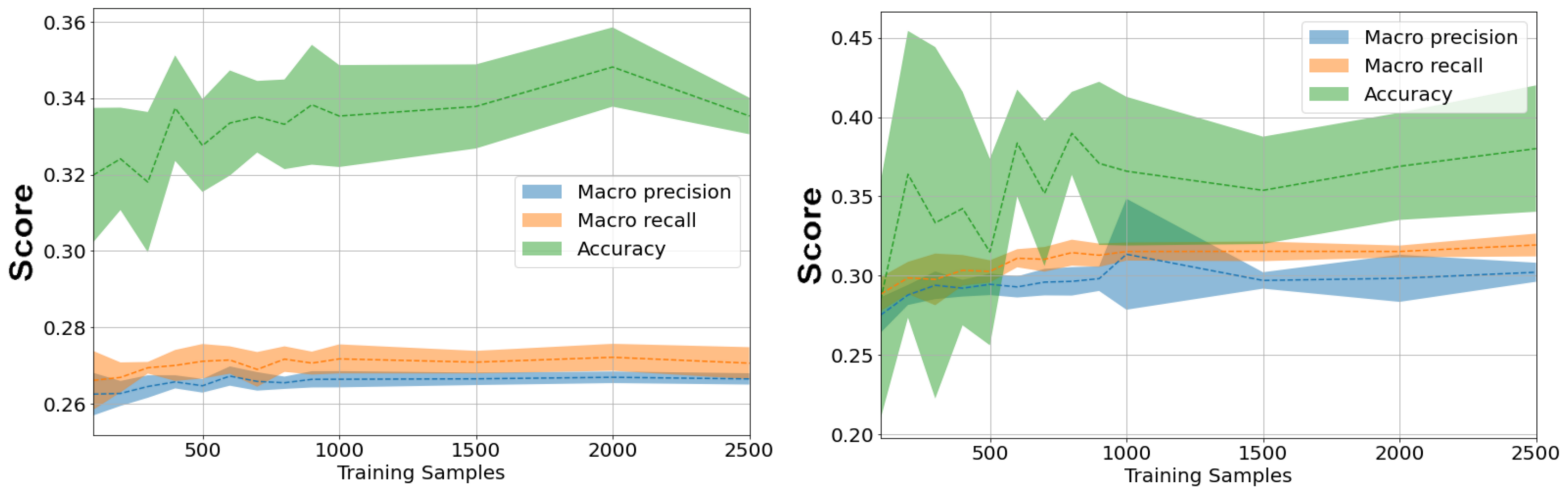
Effect of training fragments per class (100–2,500) on metagenomic taxonomy with superkingdom-balanced subsampling. Left: minimum-hit classification. Right: averaged-superkingdom classification. Curves report macro precision/recall/F1 and micro-averaged accuracy; shaded bands denote standard deviation across random subsamples. Training/testing data as in Table 1. Increasing fragments per class improves macro metrics and stabilizes classification variance.

We can see that despite a prohibitively low number of samples per class, even as few as just 100, subsampling superkingdoms achieves macro F1 scores just above Kraken2. Figure 9 show the macro/micro precision/recall of this method, as well as the standard deviation as the number of training fragments is increased.

While the overall accuracy (in this case equivalent to micro precision/recall) of superkingdom subsampling does not demonstrate any increase when compared to genome subsampling, the macro metrics do show benefits which increase alongside the volume of fragments.

**Figure 10:**
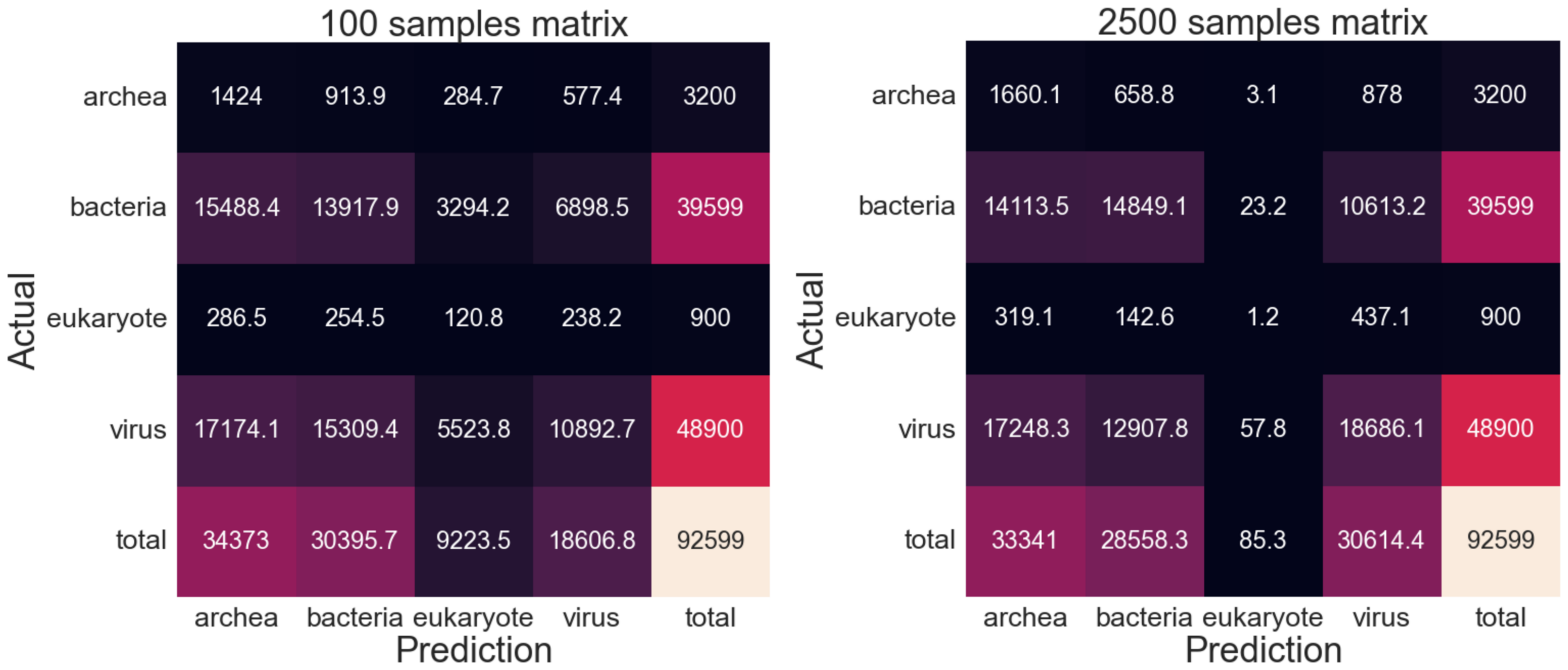
Confusion matrices for superkingdom-balanced subsampling with averaged-superkingdom classification. Left: 100 fragments/class. Right: 2,500 fragments/class. Training and test data as in Table 1. Predicted compositions more accurately approximate true proportions when balanced by superkingdom.

Confusion matrices (Fig. 10) show that although the individual predicted classes do tend to be inaccurate on a read level, the overall predicted composition of the sample seems more accurate. Predictions tend to be relatively equally distributed amongst the Archaea/Bacteria/Virus classes, with eukaryote reads predicted at a far lower rate (which is closer to the to the true composition than other NCD tests). However, there are a large portion of correct predictions for Viruses and Bacteria, although there is a confusion between those classes and Archaea. The benefit offered by increasing the number of samples is also present here, primarily seen in the decrease in errant eukaryotic predictions as the samples is increased, as well the increased proportion of virus predictions.

### Alternative compression algorithms

As noted in ‘Normalized compression distance’, any alternative compression algorithm may be used when performing NCD, and this framework is not limited to solely *gzip*. *Bzip2* is another popular compression algorithm, which utilizes the Burrows-Wheeler transform (Schindler, 1997) to first reversibly rearrange sequences into runs of similar characters, enabling higher compression ratios. *LZMA*, which is known for its impressive compression ratios (and slower compression speed) is based on a variant of LZ77 (Ziv & Lempel, 1977). It utilizes massive dictionary sizes, enabling it to handle long-distance redundancies. Table 4 compares the performance of all three compressors in the human gene classification task introduced in ‘Human DNA’, with results averaged across five runs of a 5-fold cross validation dataset.

**Table 4:** Human DNA gene classification with NCD using three compressors. Results are averaged over five runs of a 5-fold cross-validation split on the Human DNA dataset (4,380 sequences; seven classes; Section ‘Human DNA’). NCD uses minimum-hit KNN (*K* = 1). “Total Time(s)” includes both precomputing per-sequence compressed lengths and classification on each fold. Precision, recall, and F1 are macro-averaged; accuracy is micro-averaged. Results are for our hardware described in ‘Results’ (Hardware).

Method	Total Time(s)	Acc.	Prec.	Recall	F1
NCD+*gzip*	537.5	0.889	0.886	0.889	0.883
NCD+*bzip2*	984.2	0.805	0.802	0.791	0.795
NCD+*LZMA*	40,678.6	0.870	0.873	0.861	0.865

Within this specific task and setting, *gzip* attained the best total time (which includes both training and classification) and accuracy. However this does not imply universal superiority. Compressors with larger dictionaries (*e.g.*, LZMA) may be advantageous when long-range redundancy dominates; regardless this is purely speculative, and broader evaluations are warranted.

### CAMI dataset

This section focuses on analysis of the data from the CAMI II challenge, available at Fritz et al. (2021). Due to the significant computational expense of NCD-*gzip*, we analyzed only a subsample containing 10,000 reads, selected from Sample_0. This subsample is drawn from the human microbiome project dataset described by Fritz et al. (2019). Classifying these reads with our metagenomic training dataset from ‘Metagenomic reads’, using the genome-fragmented classifier described in ‘Human DNA’, produced relatively low macro-averaged scores across most taxonomic ranks (Table 5). Macro metrics were computed only over the classes present in the CAMI subsample, following standard practice. At the domain level, the dataset contains only a single true class (*Bacteria*), which explains the perfect precision but sub-maximal recall values observed there. At lower ranks, the gap between the diversity of the training set (four superkingdoms, 83 phyla) and the composition of the CAMI subsample (one superkingdom, six phyla) remains a key factor driving reduced performance.

**Table 5:** Taxonomic classification on the CAMI II 10,000-read subsample (Sample_0). Metrics are macro-averaged (recall, precision, F1) and micro-averaged (accuracy). NCD uses genome fragmentation (‘Genome fragmentation’) and assigns every read; Kraken2 uses low-confidence assignments and leaves 61.4% unclassified.

**NCD**				
Rank	Recall	Precision	F1	Acc.
Superkingdom	0.9616	1.0000	0.9804	0.9616
Phylum	0.1336	0.1637	0.1263	0.2709
Class	0.0514	0.1073	0.0543	0.1430
Order	0.0289	0.0991	0.0439	0.1178
Family	0.0098	0.0569	0.0165	0.0745

**Kraken2**				
Rank	Recall	Precision	F1	Acc.
Superkingdom	0.4023	1.0000	0.5738	0.4023
Phylum	0.5294	0.9636	0.6355	0.3961
Class	0.5722	0.8227	0.6001	0.3785
Order	0.4174	0.8734	0.4997	0.3601
Family	0.2579	0.6489	0.3102	0.2491

**Figure 11:**
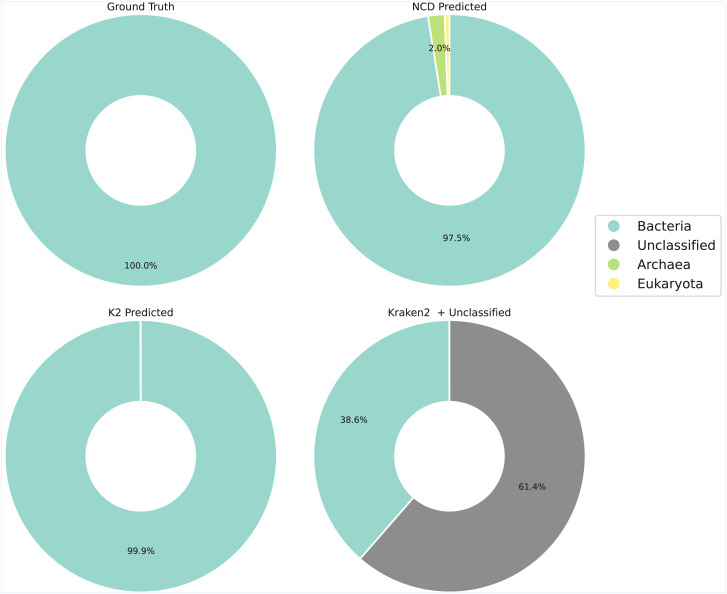
True *vs* predicted superkingdom composition for the CAMI II 10,000-read subsample. Top: NCD-*gzip* with genome fragmentation (‘Genome fragmentation’). Kraken2 results, showing classified only (left) and full predictions including unclassified reads (Right).

**Figure 12:**
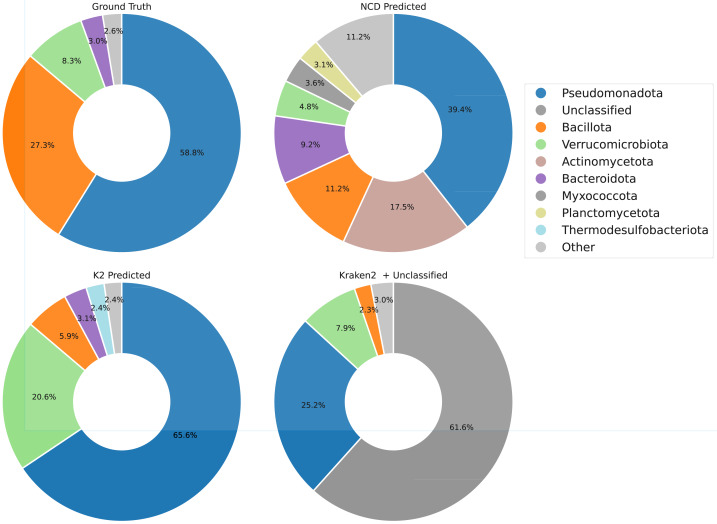
True *vs* predicted phylum composition for the CAMI II 10,000-read subsample. Top: NCD-*gzip* with genome fragmentation (‘Genome fragmentation’). Bottom: Kraken2 results, showing classified only (left) and full predictions including unclassified reads (right). “Other” combines all taxa with less than 1% representation to reduce label count.

Figures 11 and 12 show that NCD-*gzip* maintains reasonable predictive accuracy for higher-level taxa categories, although it does overestimate the presence of Actinomycetota in the sample. Kraken2, on the other hand, demonstrates consistently high precision across ranks, translating into stronger macro-averaged precision and F1 scores (Table 5). This improvement comes at the cost of recall, since a substantial proportion of reads (61.4%) were left unclassified. These unclassified reads directly reduce recall and overall sensitivity of Kraken2. In contrast, NCD-*gzip* assigns every read, yielding high recall and accuracy at the superkingdom level, but lower precision at finer taxonomic resolutions due to the broader and more diverse training label space.

Taken together, these results indicate that Kraken2 is generally stronger than NCD-*gzip* under our CAMI II evaluation. Kraken2 achieves higher macro-averaged precision and F1 scores at the lower taxanomic ranks, while NCD-*gzip* is mainly competitive when predictions are aggregated to the superkingdom level, where it benefits from assigning a label to every read. In this setting, the primary appeal of NCD-*gzip* lies in its database-agnostic, full-coverage predictions at coarse taxonomic ranks, rather than superior overall accuracy across ranks.

### Timing results

A potential downside of NCD is the significant computational requirements of classification, especially as the volume of data is increased. Table 6 shows this clearly, with significant classification times for metagenomic samples especially when compared to Kraken2 (Table 7). The bottleneck arises from the pairwise compression step, which must be repeated for each test–train sequence pair in order to compute NCD. As the number of training genomes and reads increases, this results in quadratic scaling of runtime and memory demand, making the approach challenging to apply at scale without considerable parallelization or approximation strategies.

Language model baselines (HyenaDNA, CADUCEUS) were used in their pretrained form in our experiments, and thus their specific training resources are unknown. MetaBERTa, however, was trained our group, and required approximately 29 h on four V100 GPUs (totaling ∼26GB memory usage).

## Conclusion: discussion and future work

In this paper, we have demonstrated that the combination of NCD+*gzip* as developed by Jiang et al. (2023) is capable of reasonable classification accuracy in both gene and taxonomy classification. It is especially adept at distinguishing related human genes compared to all other methods, including a previous implementation of NCD. For prokaryotic gene classification and metagenomics, it rivals state of the art classifiers such as Kraken2 and MMseqs2 for the superkingdom level, and unlike taxonomic classifiers, it can also identify gene label, albeit with less performance than alignment. The higher-level taxonomic improvement can be attributed to ability of *gzip*, and by extension NCD, to take into consideration diverse features of each sequence, including variable length k-mer/nucleotide frequency, sequence length, and repetitiveness to classify. This enables NCD to perform reasonably well across tasks, balancing the capabilities of both short and long sequence matches to detect identifying biological signals despite withheld representation. This underscores the capacity of NCD to generalize well, often performing more closely with ML models than to more traditional alignment-free classification techniques.

**Table 6:** NCD-*gzip* single-threaded training and classification timing results. The significant memory usage in the RefSeq Prokaryote tasks stems from the size of the distance matrix. In later runs, this array was not fully stored in memory; instead, only *K*-minimum distance hits were retained.

Database	Subsampling parameters	Training database		Training		Subset	Testing database		Classify	
		Seq #	Size (MB)	Time	Mem (GB)		Seq #	Size (MB)	Time	Mem (GB)
**Human DNA**	–	2,629	3.33	0.41s	0.034	Test	1,314	1.67	9 m 6.6 s	0.034
**RefSeq Prokaryote**	–					Taxa_out	11,800	12.51	47.29 h	52.46
		547,523	588	40s	1.40	Gene_out	43,417	48.16	821 h	191.64
						Test	140,524	150.66	2862 h	889.0
**Metagenome**	All fragments		11,610	15 m 3 s	14.78	Genome fragmented			173053 h	7.1
	1/2,000*all_frags	3,708 genomes	5.81	8 m 28 s	4.79		92,600 reads	13.54	35.09 h	4.79
	1/4,000*all_frags		2.90	6 m 49 s	1.36				20.83 h	1.36
	500 frags/sk	2,000 frags	0.237	1 m 22 s	2.89	Superkingdom (sk) Fragmented			0.916 h	2.89
	1,000 frags/sk	4,000 frags	0.475	1 m 24 s	2.66		92,600 reads	13.54	1.855 h	2.66
	2,000 frags/sk	8,000 frags	0.950	1 m 30 s	2.84				3.837 h	2.84

**Table 7:** Kraken2 metagenomic single-threaded read classification timing results. Training is performed in two sequential steps: *add-to-lib* and *build-db*.

Tool	Testing Seq #	Test size (MB)	Train seq #	Train Size (MB)	Training Time		Training Mem (GB)		Classify time	Classify Mem (GB)
					Add-to-lib	Build-db	Add-to-lib	Build-db		
Kraken2	92,600 reads	13.54	3,708 genomes	11,610	1 h 56 m	38 m	0.084	13.71	1.386s	7.85

However, the computational requirements for this method can be prohibitive and are subject to the compression rate of *gzip* on a given machine. Simple alignment-free sequence comparison methods which rely on comparing each sequence to all elements in a database simply do not scale well, especially as learned methods become more reliable. Despite this, a primary strength of NCD+*gzip* +KNN is that it functions with low requirements for training (only the individual database sequence compression length need to be computed, which is relatively fast). This is a particular advantage for smaller datasets, such as the human gene dataset presented by Ali et al. (2024). In these sorts of tasks, with representative sequences but a smaller database, NCD is capable of high performance comparable to other state of the art tools. NCD is similarly powerful for datasets with limited representation, where its strong generalization performance enables classification accuracy that can compete with and even exceed GLM-based methods. Furthermore this implementation of NCD is both simple and adaptable. Here we have demonstrated efficacy in gene and taxonomy labeling, and can likely be easily applied to other tasks such as promoter detection and antimicrobial resistance prediction. Additionally, NCD requires relatively few hyperparameters, avoiding the need for complicated setup procedures and tuning.

In the future, there are several areas for improvements. A simple inclusion would be a confidence metric, which could be leveraged to improve the precision of NCD. There is also room for research into different compression algorithms, which can be readily swapped into the program. Another important limitation of the fragmentation approaches introduced in this paper is its dependence on the expected test sequence length. Because we rely on fragmenting the training database into sequences of comparable length to the test data, a change in read length between training and deployment requires re-fragmentation of the database. This reduces the generalizability of the method across different sequencing settings. Future directions include developing length-normalization strategies, or length-robust compression schemes, which would allow a single trained database to support a wider range of test sequence lengths. Ultimately NCD+*gzip* +KNN, while an incomplete tool, presents a functional method for genomic analysis, with superior generalization despite its minimal complexity, as well as a clear path for future development.

## Additional Information and Declarations

### Data Availability

The following information was supplied regarding data availability: The code is available at GitHub and Zenodo: - https://github.com/ghproducts/genomics-ncd-gzip/tree/main
- ghproducts. (2025). ghproducts/genomics-ncd-gzip: Initial publication release (v1.0.0). Zenodo. https://doi.org/10.5281/zenodo.17204161
The Human DNA Data is available at Kaggle: nageshsingh/dna-sequence-dataset The Metagenomic Dataset is available at Zenodo: EESI lab. (2025). Genomics NCD-gzip database [Data set]. Zenodo. https://doi.org/10.5281/zenodo.15635653
The Gene-Taxa Dataset is available at Zenodo: Refahi, M. S. (2024). Scorpio Gene-Taxa Benchmark Dataset [Data set]. Zenodo. https://doi.org/10.5281/zenodo.12964684.
